# Effect of Geometric and Chemical Anisotropy of Janus Ellipsoids on Janus Boundary Mismatch at the Fluid–Fluid Interface

**DOI:** 10.3390/ma9080664

**Published:** 2016-08-06

**Authors:** Dong Woo Kang, Woong Ko, Bomsock Lee, Bum Jun Park

**Affiliations:** Department of Chemical Engineering, Kyung Hee University, Yongin, Gyeonggi-do 17104, Korea; lukekang070@gmail.com (D.W.K.); wng55555@gmail.com (W.K.); bslee@khu.ac.kr (B.L.)

**Keywords:** Janus particle, fluid–fluid interface, attachment energy, configuration, Janus boundary

## Abstract

We investigated the geometric and chemical factors of nonspherical Janus particles (i.e., Janus ellipsoids) with regard to the pinning and unpinning behaviors of the Janus boundary at the oil–water interface using attachment energy numerical calculations. The geometric factors were characterized by aspect ratio (*AR*) and location of the Janus boundary (*α*) separating the polar and apolar regions of the particle. The chemical factor indicated the supplementary wettability (*β*) of the two sides of the particle with identical deviations of apolarity and polarity from neutral wetting. These two factors competed with each other to determine particle configurations at the interface. In general, the critical value of *β* (*β_c_*) required to preserve the pinned configuration was inversely proportional to the values of *α* and *AR*. From the numerical calculations, the empirical relationship of the parameter values of Janus ellipsoids was found; that is, $\lambda =\Delta \beta_{c}/\Delta \alpha \approx 0.61AR-1.61$. Particularly for the Janus ellipsoids with *AR* > 1, the *β_c_* value is consistent with the boundary between the tilted only and the tilted equilibrium/upright metastable region in their configuration phase diagram. We believe that this work performed at the single particle level offers a fundamental understanding of the manipulation of interparticle interactions and control of the rheological properties of particle-laden interfaces when particles are used as solid surfactants.

## 1. Introduction

Typical colloidal particles with appropriate wettability can be irreversibly adsorbed at fluid–fluid interfaces (e.g., oil–water and air–water interfaces) [1,2]. Strong particle adsorptions can decrease interfacial tension and stabilize the interface, thereby preventing phase separation and coalescence in emulsion systems. In particular, chemically homogeneous colloidal particles likely impart kinetic stability in Pickering emulsions stabilized by solid particles [3,4]. The efficacy of interfacial stability is highly dependent on the relative amount of each fluid phase and particle wettability (polarity or apolarity) with regard to the fluid–fluid interface that can be characterized via three-phase contact angles.

Janus particles that possess chemical and/or geometric anisotropy can improve the stabilization efficiency of Pickering emulsion systems when used as stabilizers [5,6,7,8,9,10,11,12,13,14,15]. This is because Janus particles tend to be aligned toward increasing the surface area of preferred wetting (e.g., apolar surfaces exposed to oil and polar surfaces exposed to water), resulting in effectively reduced surface free energy. Additional degrees of freedom gained from anisotropic properties can lead to diversity in configurational behaviors (upright or tilted) when attached to a fluid–fluid interface [16,17,18,19,20]. It is important to note that configurations on the single-particle level can significantly affect interactions between particles at the interface, their assembly structures, and consequently the rheological properties of the interface [21,22,23,24,25,26,27,28,29,30,31]. For instance, Janus spheres with two chemically different sides (apolar and polar surfaces) generally adopt an upright orientation corresponding to the geometry in which the Janus boundary or wettability separating line (WSL) is pinned to the fluid–fluid interface [32]. With regard to non-spherical Janus particles (e.g., Janus ellipsoids, Janus dumbbells, and Janus cylinders), particle configurations can be either upright or tilted, depending on the relative influence of chemical and geometric factors. Particularly, particles adopted tilted configurations when the geometric effect (e.g., aspect ratio, *AR*) is stronger than the chemical effect (e.g., wettability), whereas particles under the opposite conditions adopted upright configurations. Janus particles with tilted configurations likely deformed their surrounding fluid interface due to unpreferred wetting (e.g., apolar surfaces exposed to water, and vice versa), and the resulting interface deformation led to lateral capillary interactions between particles to minimize the surface areas of the deformed interfaces [33,34,35,36,37,38,39]. Capillary interactions between particles at the interface directly affected their microstructure, and therefore the rheological properties of the particle-laden interface. Effects of configuration at the single-particle level on interparticle interactions were experimentally demonstrated using Janus cylinders with apolar and polar surfaces prepared via PDMS (Polydimethylsiloxane) molding techniques [33,34,40]. Apolar and polar precursor solutions were added to a PDMS mold with cylindrical wells one after another, and subsequent UV exposure led to photopolymerization of the precursor solution, resulting in Janus cylinders. The aspect ratio and relative lengths of the two sides could be readily controlled depending on the well depth in the mold and the amount of each precursor solution. Janus cylinders with high *AR* values adopted tilted configurations and exhibited strong capillary attractions. In contrast, low *AR* particles adopted upright configurations and attractive interactions occurred negligibly.

The configurational behaviors of two types of non-spherical Janus particles (i.e., Janus ellipsoids and Janus dumbbells) have been theoretically investigated by numerically calculating the attachment energy at the fluid–fluid interface as a function of orientation angle [16,17]. With regard to Janus dumbbells, a single energy minimum occurred in the attachment energy profile, indicating that the Janus dumbbells exclusively adopted either an upright or a tilted configuration. Interestingly, for Janus ellipsoids with particular *AR* and wettability values, two energy minima appeared in the attachment energy profile; one corresponding to an upright configuration, and the other to a tilted configuration—one of the two configurations should be kinetically stable. With regard to Janus ellipsoids, it is unknown whether the Janus boundary of the particles can be detached or unpinned from the fluid–fluid interface, regardless of configuration. These pinning and unpinning behaviors might depend on the relative effect of chemical and geometric anisotropy. Indeed, it was previously reported that the Janus boundary of Janus spheres could be unpinned from the interface when the boundary significantly deviated from the central region of the particles [2,32]. In this study, we quantitatively investigate the effects of geometric and chemical factors of Janus ellipsoids on the pinning and unpinning behaviors of the Janus boundary at the fluid interface. We also relate the unpinned configuration to the tilted configuration when the *AR* value is larger than 1.

## 2. Theoretical Basis

**Figure 1:**
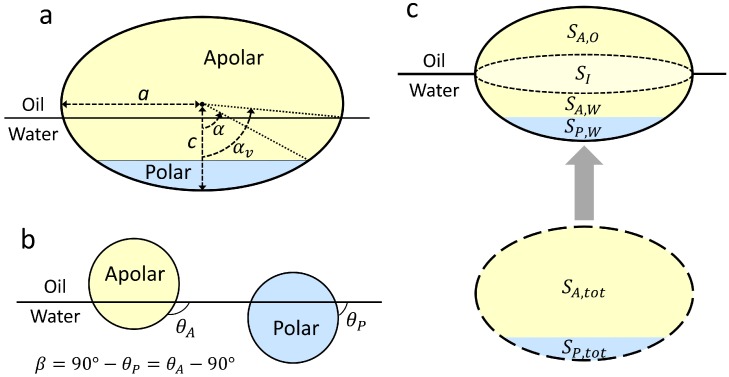
Schematics of a Janus ellipsoid for attachment energy calculations. (**a**) Geometric relationships of the Janus ellipsoid; (**b**) Three-phase contact angles of homogeneous polar and apolar spheres at the oil–water interface; (**c**) Schematic illustration of Janus ellipsoid attachment to the oil–water interface.

To characterize the geometric and chemical anisotropy of Janus ellipsoids, we define the aspect ratio (*AR*), the location of the Janus boundary (*α*), and the supplementary wettability (*β*) as shown in Figure 1a,b. The aspect ratio is the ratio of the major axis to the minor axis, calculated by *AR* = *c*/*a*. Depending on the value of *AR*, the ellipsoids are prolate or oblate at *AR* > 1 and *AR* < 1, respectively, with a sphere formed at *AR* = 1. We designate all studied particles as Janus ellipsoids. The Janus boundary that indicates the wettability separation line (WSL) dividing polar and apolar regions is characterized by an elevation angle, *α*. The supplementary wettability is defined as $\beta =90{}^{\circ}-\theta_{P}=\theta_{A}-90{}^{\circ}$, in which $\theta_{P}$ and $\theta_{A}$ indicate the three-phase contact angles of homogeneous polar (*α* = 180°) and apolar (*α* = 0°) spheres when trapped at an oil–water interface, respectively (Figure 1b).

Based on these geometric relationships, the attachment energy of a particle that is attached to a planar oil–water interface from the water phase (Figure 1c) is given by [1,2,16,17]

(1) $$\Delta E_{Iw}=E_{I}-E_{w}$$

The respective energies of a particle at the oil–water interface (*E_I_*) and a particle immersed in the aqueous phase (*E_w_*) are

(2) $$E_{I}=S_{Ao}\gamma_{Ao}+S_{Aw}\gamma_{Aw}+S_{Po}\gamma_{Po}+S_{Pw}\gamma_{Pw}+S_{I}^{\left(2\right)}\gamma_{ow}$$

(3) $$E_{w}=S_{A}^{tot}\gamma_{Aw}+S_{P}^{tot}\gamma_{Pw}+S_{I}^{\left(1\right)}\gamma_{ow}$$

where *S_ij_* is the surface area (*i* = *P* (polar) or *A* (apolar)) exposed to a fluid phase (*j* = *w* (water) or *o* (oil)), *γ_ij_* is the corresponding surface energy of surface *i* and fluid *j*, $S_{I}^{\left(2\right)}$ and $S_{I}^{\left(1\right)}$ are the surface area values of the oil–water interface when particles are present and absent, respectively, and $S_{I}=S_{I}^{\left(2\right)}-S_{I}^{\left(1\right)}$ indicates the displaced area of the interface due to presence of the particle. The displaced area (*S_I_*) corresponds to the cross-sectional area of the particle at *α_v_*, which is the elevation angle measured from the major axis to the fluid interface (i.e., the three phase contact line), as shown in Figure 1a. $S_{A}^{tot}$ and $S_{P}^{tot}$ are the total surface area values of the apolar and polar regions of the particle, respectively. Substitution of the following Young’s Equations:

(4) $$\gamma_{ow}\cos\theta_{P}=\gamma_{\text{Po}}-\gamma_{Pw}\;\text{for the polar surface}$$

(5) $$\gamma_{ow}\cos\theta_{A}=\gamma_{Ao}-\gamma_{Aw}\;\text{for the apolar surface}$$

into Equations (1)–(3) yields the following:

(6) $$\Delta E_{Iw}=\gamma_{ow}\left(S_{Ao}\cos\theta_{A}+S_{Po}\cos\theta_{P}-S_{I}\right)\;\text{from the water phase}$$

Similarly, the attachment energy of a Janus particle from the oil phase to the oil–water interface can be expressed as:

(7) $$\Delta E_{Io}=-\gamma_{ow}\left(S_{Aw}\cos\theta_{A}+S_{Pw}\cos\theta_{P}+S_{I}\right)\;\text{from the oil phase.}$$

The difference between Equations (6) and (7) is the reference energy state (i.e., *E_w_* and *E_o_*); therefore, the shapes of the attachment energy profiles obtained from the equations should be identical. Since the use of either Equation (6) or (7) predicts the same configurational behavior, Equation (6) is used in this work. To calculate the surface area *S_ij_*, a numerical method using the hit-and-miss Monte Carlo method is employed [16]. The oil–water interfacial tension value is arbitrarily designated as *γ_ow_* = 50 mN/m, corresponding to the interfacial tension of a decane–water interface.

In the numerical calculations, it is assumed that the meniscus at the particle interface is smooth. The effect of line tension at the three-phase contact line can be neglected when the particle dimensions are sufficiently large (e.g., >10 nm) [15,41]. In this case, the effect of the Brownian motion of particles on the configuration behaviors is also negligible [16]. The oil–water interface around the particle is assumed to be flat when the particle size is less than a few hundred microns, in which the corresponding Bond number (ratio of gravitational force to surface tension force) is sufficiently small. Although interfacial deformation around the particles is possible for non-spherical Janus particles, it was previously demonstrated that the flat interface assumption (FIA) with regard to the attachment energy calculations was appropriate to determine the equilibrium configurations of the particles at the fluid–fluid interface [16]. Moreover, it was reported that the configuration behaviors of Janus cylinders predicted from the numerical calculations based on the FIA showed a good agreement with the experimental observations [33,34]. Note that in spite of the consistency between the experimental and theoretical results for the configuration behaviors when the FIA is used, interfacial deformation around Janus particles with a tilted configuration can occur, leading to capillary-induced interactions between the tilted Janus particles [33,34,35]. Based on these conditions and methods, the attachment energy is calculated as a function of vertical position, *α_v_*. To evaluate whether the Janus boundary of the particles is pinned or unpinned at the oil–water interface, the attachment energy is minimized to obtain the lowest energy minimum, $\Delta E_{att}^{min}\left(\alpha_{v}\right)$, and the corresponding vertical position , *α_v,min_*.

Notably, for Janus prolates with *AR* > 1, there are four possible cases in their configuration behaviors, depending on *AR* and wettability values, such as the upright only, the upright equilibrium/tilted metastable, the tilted equilibrium/upright metastable, and the tilted only regions [16,17]. For the co-existing regions, for instance, two energy minima appear in the attachment energy profiles, indicating that particles can adopt either upright or tilted configurations, whereas particles in the titled only region possess only the tilted configuration. Therefore, to further determine the configuration behaviors (upright or/and tilted) of the Janus prolates, the attachment energy is calculated as functions of the vertical position (*α_v_*) as well as the orientation angle (0° < *θ_r_* < 90°), in which *θ_r_* = 0° and 90° indicate the geometries of the Janus boundary parallel and perpendicular to the interface, respectively. In this case, the attachment energy is scanned by varying the *α_v_* values at a constant *θ_r_*. The minimum attachment energy is then calculated at the given *θ_r_* value, and the same procedure is repeated for different values of *θ_r_* to find a global energy minimum, $\Delta E_{att}^{min}\left(\alpha_{v},\theta_{r}\right)$.

For spherical Janus particles with supplementary wettability (i.e., $\cos\theta_{A}=-\sin\beta$ and $\cos\theta_{A}=\sin\beta$), the attachment energy from the water phase (Equation (6)) can be further simplified using geometric relationships. In particular, when the Janus boundary of a Janus sphere is pinned at the oil–water interface, the surface areas of the apolar and polar regions exposed to the oil phase are $S_{Ao}=2\pi a^{2}\left(1+\cos\alpha \right)$ and $S_{Po}=0$, respectively. The displaced area in this geometry is $S_{I}=\pi a^{2}\sin^{2}\alpha$. Therefore, Equation (6) becomes,

(8) $$\Delta E_{Iw}=-\pi a^{2}\gamma_{ow}\left(2\left(1+\cos\alpha \right)\sin\beta +\sin^{2}\alpha \right)\;\text{from the water phase}$$

Equation (8) should satisfy the condition of $\frac{d\left(\Delta E_{Iw}\right)}{d\alpha }=0$ at $\beta =\beta_{c}$ for the Janus sphere with the pinned Janus boundary, yielding $\sin\beta_{c}=\cos\alpha$, and thus $\beta_{c}=90{}^{\circ}-\alpha$.

## 3. Results and Discussion

Pinning (*α* = *α_v,min_*) and unpinning (*α* ≠ *α_v,min_*) behaviors of the Janus particles with respect to the interfaces can be determined through two competing factors: geometric and chemical anisotropy. To minimize the attachment energy in Equation (6), a Janus particle tends to be aligned toward increasing the displaced area (*S_I_*) at the oil–water interface, simultaneously increasing the surface area of the preferred wetting state (*S_Ao_*; note that $\cos\theta_{A}<0$). The state of preferred wetting indicates the configuration of the apolar and polar regions exposed to their favorable fluid phases, oil and water, respectively. Depending on the relative influence of these two factors, the particle is likely to preferentially adopt the pinned configuration when wettability effects are greater than geometric effects, whereas particles are unpinned from the interface when geometric effects are dominant. Notably, Janus prolates can adopt the two configurations, the upright and tilted one (e.g., when the *AR* value is high and wettability is moderate) [16]. In this particular case, the attachment energy may be further decreased by rotating the particle at the interface. We also examine the relationship between the pinning/unpinning and the upright/tilted configuration behaviors.

To investigate the effects of geometry (*AR* and *α*) and wettability (*β*) on the pinning and unpinning behaviors of Janus ellipsoids, the attachment energy ($\Delta E_{Iw}$) is calculated as a function of *α_v_* at constant values of *β* and *AR* = 0.5 (oblate), 1 (sphere), or 2 (prolate). Since the supplementary wettability (*β*) is used and Janus particles with *α* ≤ 90° are considered, $\Delta E_{Iw}$ is calculated in the range of $0{}^{\circ}\leq \alpha_{v}\leq 90{}^{\circ}$. Note that similar results can be obtained in the case of Janus ellipsoids with *α* > 90°.

**Figure 2:**
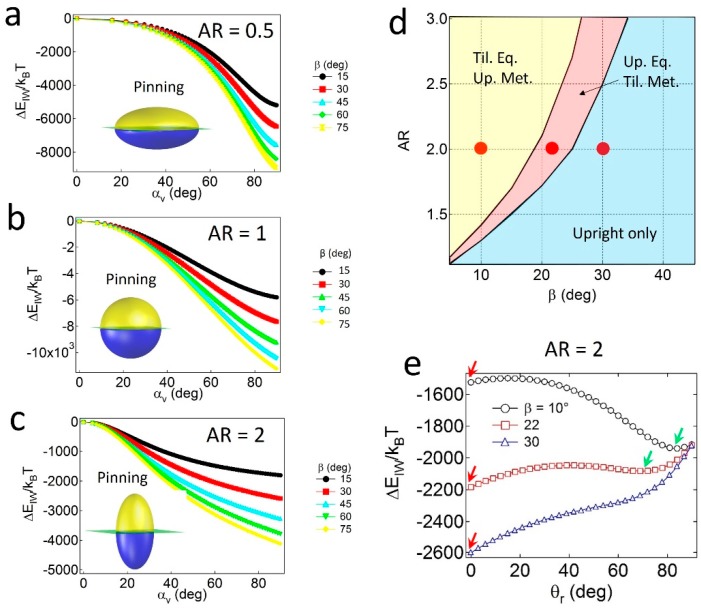
Attachment energy profiles of Janus ellipsoids with *α* = 90° and (**a**) Aspect ratio (*AR*) = 0.5 (oblate); (**b**) 1 (sphere); or (**c**) 2 (prolate). In all cases, the Janus boundary is pinned at the oil–water interface; (**d**) Configuration phase diagram of Janus prolates as functions of the *AR* and *β* values; (**e**) The attachment energy profiles of Janus prolates as a function of the orientation angle (*θ_r_*).

For the Janus ellipsoids with *α* = 90°—in which the polar and apolar surface area values are identical—the lowest energy minimum ($\Delta E_{att}^{min}$) for all cases is found at *α_v,min_* = 90°, as shown in Figure 2a–c. The result of *α* = *α_v,min_* indicates that the Janus boundary of the geometrically symmetric Janus ellipsoids is always pinned at the oil–water interface, regardless of the values of *AR* and *β*. The pinned configuration of the particles simultaneously satisfies both conditions; that is, the particles tend to possess maximum values of the displaced area (*S_I_*) and preferred wetting (*S_Ao_*) in Equation (6) when *α* = *α_v,min_*.

Janus prolates with particular *AR* and *β* values can also adopt the tilted configuration. As shown in the configuration phase diagram in Figure 2d, particles with relatively high *β* and low *AR* values only possess the upright or the pinned configuration (light blue region), whereas two coexisting regions (pink and yellow regions) appear when particles carry the opposite properties (i.e., relatively low *β* and high *AR* values). For instance, for particles with *AR* = 2 and *β* = 10° or 22° (denoted as red dots in Figure 2d), two energy minima exist in the attachment energy profiles in Figure 2e; that is, one corresponds to the upright (red arrow) and the other to the tilted one (green arrow). In contrast, a single energy minimum for the particle with *AR* = 2 and *β* = 30° (Figure 2e) indicates that the particle solely adopts the upright configuration. Notably, the absence of a tilted only region in Figure 2d might be related to the absence of the unpinned configuration. In other words, since the Janus boundary of the particles with *α* = 90° can always be pinned to the interface, regardless of the values of *AR* and *β*, at least one of the energy minima in the attachment energy profiles should exist at *θ_r_* = 0.

**Figure 3:**
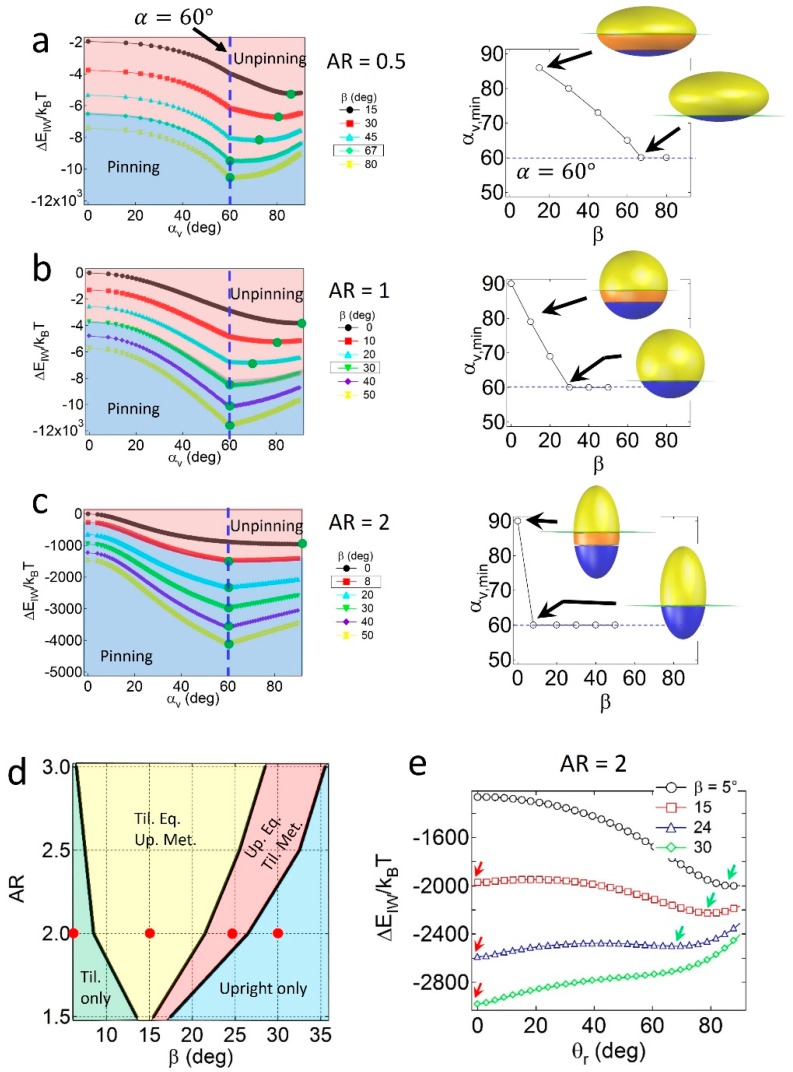
Attachment energy profiles of Janus particles with *α* = 60° (vertical dashed line) and (**a**) *AR* = 0.5; (**b**) 1; or (**c**) 2. Pink and blue regions in each plot represent unpinned and pinned configurations, respectively. Green circles denote the lowest energy minimum ($\Delta E_{att}^{min}$) and the corresponding value of *α* (*α_v,min_*). The effect of *β* on *α_v,min_* is shown on the right; (**d**) Configuration phase diagram of Janus prolates as functions of the *AR* and *β* values; (**e**) The attachment energy profiles of Janus prolates as a function of the orientation angle (*θ_r_*).

When the particles are geometrically asymmetric (*α* = 60°), configuration behavior depends on the relative geometry and wettability effects. As shown in Figure 3a, for oblates with *AR* = 0.5, the value of *α_v,min_* at $\Delta E_{att}^{min}$ gradually decreases with *β*, and is not consistent with *α* if the value of *β* is not sufficiently high. The condition of *α_v,min_* = *α* indicates that a pinned geometry is found at a critical value of *β*, in which *β_c_* = 67°. Similar results are found for spheres with *AR* = 1 (Figure 3b) and prolates with *AR* = 2 (Figure 3c) when *α* = 60°, in which the critical wettability values are *β_c_* = 30° and 8°, respectively. The increase in *β_c_* for lower *AR* particles is due to the relatively large displaced area (*S_I_*) around the central regions of the particles; thus, the geometric effects become stronger than the wettability effects. Note that the value of *β_c_* = 30° for the Janus sphere obtained from the numerical calculation is consistent with the theoretical prediction of $\beta_{c}=90{}^{\circ}-\alpha$, validating the numerical method.

The configuration behaviors of geometrically asymmetric Janus prolates are also affected by the presence of the secondary energy minimum in the attachment energy profile. As shown in the configuration phase diagram of Janus prolates with *α* = 60° in Figure 3d, four distinct regions are observed; the tilted only (light green), the tilted equilibrium/upright metastable (yellow), the upright equilibrium/tilted metastable (pink), and the upright only (light blue) regions. The example attachment energy profiles as a function of *θ_r_* are shown in Figure 3e, and the corresponding energy minima are indicated by red and green arrows. Unlike the Janus prolates with *α* = 90°, it is interesting to note that the tilted only region is found at the conditions of relatively high *AR* and low *β* values. The presence of the tilted only region is likely due to the presence of the unpinning configuration of the particles; that is, when the prolate particles are unpinned, no energy minimum exists at *θ_r_* = 0, and consequently, the particles only adopt the tilted configuration. In contrast, when the Janus boundary of prolate particles are pinned to the interface in Figure 3c, they essentially possess an energy minimum at *θ_r_* = 0, leading to the upright/tilted coexisting regions (yellow and pink areas in Figure 3d) or the upright only region (light blue in Figure 3d). Note that the *β_c_* = 8° value for the Janus prolate with *AR* = 2 in Figure 3c shows a good agreement with the boundary between the light green and yellow regions in the Figure 3d.

**Figure 4:**
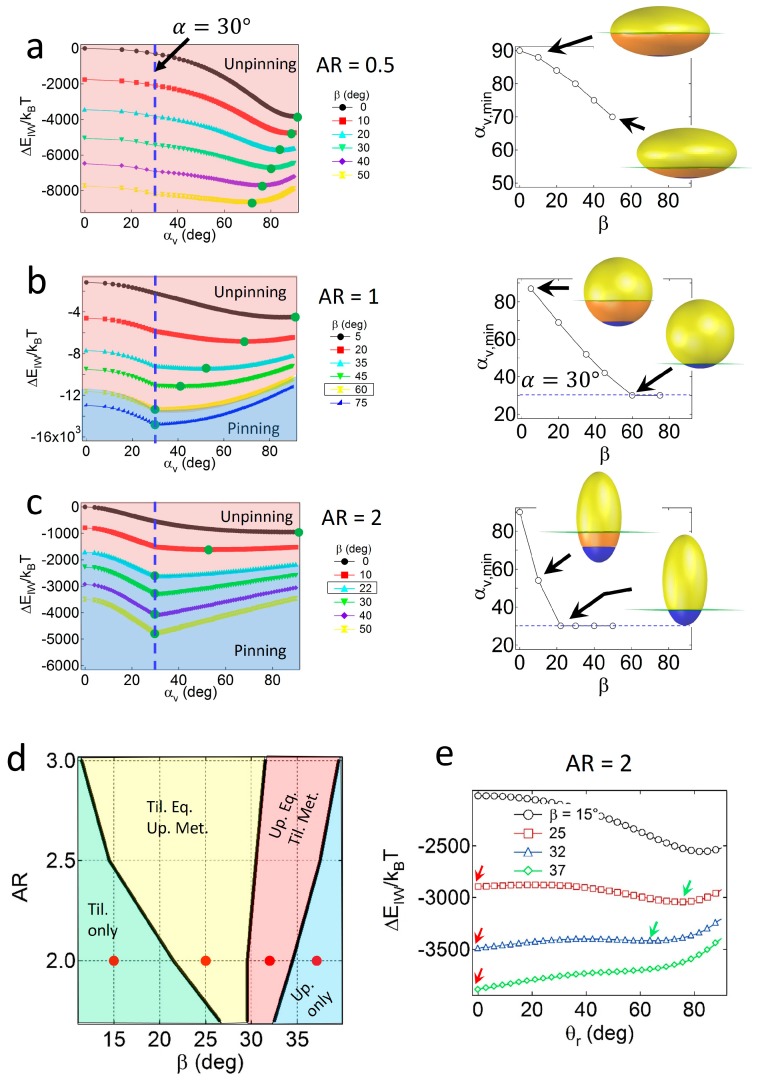
Attachment energy profiles of Janus particles with *α* = 30° (vertical dashed line) and (**a**) *AR* = 0.5; (**b**) 1; or (**c**) 2. Green circles denote the values of $\Delta E_{att}^{min}$ and *α_v,min_*. The relationship between *β* and *α_v,min_* is shown on the right; (**d**) Configuration phase diagram of Janus prolates as functions of the *AR* and *β* values; (**e**) The attachment energy profiles of Janus prolates as a function of the orientation angle (*θ_r_*).

For Janus ellipsoids with a high degree of geometric asymmetry (*α* = 30°), strong wettability effects are required to preserve the pinned configuration. As shown in Figure 4a, for oblates with *AR* = 0.5, the value of *α_v,min_* consistently decreases as *β* increases, and does not match the value of *α* = 30° up to *β* = 50°. Due to large values of *S_I_* around the central regions of the oblate, geometric effects are likely to be dominant to wettability effects, resulting in particles with central regions located at the interface, adopting an unpinned configuration. For Janus ellipsoids with lower *AR* values, wettability effects gradually become significant. As shown in Figure 4b,c, the Janus boundaries of Janus ellipsoids with *AR* = 1 and 2 are pinned to the interface when the critical wettability value is *β_c_* = 60° or 22°. Note that the value of *β_c_* increases as the particles become more geometrically asymmetric (*β_c_* = 30° and 8° for particles (*α* = 60°) with *AR* = 1 and 2, respectively).

Similar to the case of the Janus prolates with *α* = 60°, the four distinct regions in the configuration phase diagram for the prolate particles with *α* = 30° are found, as shown in Figure 4d. Example attachment energy profiles as a function of *θ_r_*, representing each configuration region and the location of energy minima, are shown in Figure 4e. The tilted only region (light green area) in relatively high *AR* and low *β* values corresponds to the condition where the Janus boundary of particles is unpinned. For particles with *AR* = 2, for instance, the boundary between the light green and the yellow regions in Figure 4e is consistent with the critical wettability value, *β_c_* = 22° in Figure 4c. Except for the tilted only region, a portion of the particles always adopts the upright configuration due to the presence of an energy minimum at the pinned geometry in Figure 4c.

**Figure 5:**
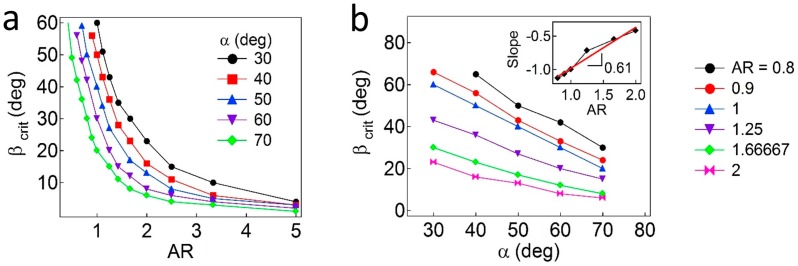
Critical wettability values (*β_c_*) as functions of *AR* and *α*. (**a**) Plot of *β_c_* versus *AR*. The regions above and below each curve correspond to the pinned and unpinned configurations, respectively; (**b**) Plot of *β_c_* versus *α*. The inset indicates the slope of each line in the plot (*β_c_* versus *α*) as a function of *AR*.

Additionally, the critical values of *β* are further calculated as functions of *AR* and *α*. As shown in Figure 5a, the regions above each curve correspond to the pinned Janus boundary at the interface, whereas the regions below the curve indicate that the Janus boundary is unpinned from the interface. In general, the value of *β_c_* (due to wettability effects) is inversely proportional to the values of *α* and *AR*, which are due to geometric effects. As the values of *AR* decrease, the particles demonstrate reduced attachment energy when the interface is located at the central regions of the particles. When the value of *α* decreases (the particles become more geometrically asymmetric), a higher value of *β* is required to adopt a pinned configuration. More quantitatively, to find a relationship of *β_c_*, *α*, and *AR*, the *β_c_* values are plotted as a function of *α*, as shown in Figure 5b. Then, slopes ($\lambda =\Delta \beta_{c}/\Delta \alpha$) obtained from linear regression of each line representing different *AR* values are shown in the inset plot in Figure 5b, in which the slope (Δλ/ΔAR) is found to be ~0.61 ± 0.06. Based on the values of λ=−1 when *AR* = 1, an empirical relationship for Janus ellipsoids is obtained, $\lambda =\Delta \beta_{c}/\Delta \alpha \approx 0.61AR-1.61$.

Finally, it is notable that when the Janus boundary of Janus prolates (*AR* > 1) with *β* < *β_c_* is unpinned, they rotate and adopt the tilted configuration to further decrease their attachment energy by increasing the displaced area (*S_I_*) [16,17] and, consequently, all portions of the particles would adopt the tilted configuration. In this case, the *β_c_* value corresponds to the boundary between two configuration regions—the tilted only and the tilted equilibrium/upright metastable regions.

## 4. Conclusions

Pinning and unpinning behaviors of the Janus boundaries of Janus ellipsoids at the oil–water interface were quantitatively investigated using numerical calculations of the attachment energy. Particle configurations were determined via two competing factors: geometric (*α* and *AR*) and chemical (*β*) anisotropy values. For geometrically and chemically symmetric Janus ellipsoids (*α* = 90°), the Janus boundary was always pinned to the interface, regardless of the values of *AR* and *β*. Contrarily, for geometrically asymmetric Janus ellipsoids with *α* ≠ 90°, the Janus boundary was unpinned when chemical effects (*β*) were not sufficiently high. In general, particles with large values of *α* and *AR* required stronger wettability to preserve the Janus boundary pinned to the interface. It was also found that determination of the critical values of *β* as functions of *α* and *AR* yielded the empirical relationship $\lambda =\Delta \beta_{c}/\Delta \alpha \approx 0.61AR-1.61$. It was notable that for the Janus prolates, the value of *β_c_* corresponded to the boundary between the tilted only and the tilted equilibrium/upright metastable regions in their configuration phase diagram. In the case of non-supplementary wettability where $90{}^{\circ}-\theta_{P}\neq \theta_{A}-90{}^{\circ}$, the pinning and unpinning behaviors are likely similar to the case of supplementary wetting when the orientation angle is not considered (*AR* ≤ 1). For Janus prolates that can adopt tilted configurations, it was reported that the relative strength of *θ_P_* and *θ_A_* significantly affected the tilted angle and the configuration behaviors [17]. We believe that this work offers fundamental ideas with regard to the attachment and configurational behaviors of nonspherical Janus particles that consequently enable manipulation of interparticle interactions and control of rheological properties of interfaces when used as solid surfactants.
